# Optimal linkage disequilibrium splitting

**DOI:** 10.1093/bioinformatics/btab519

**Published:** 2021-07-14

**Authors:** Florian Privé

**Affiliations:** National Centre for Register-Based Research, Aarhus University, Aarhus 8210, Denmark

## Abstract

**Motivation:**

A few algorithms have been developed for splitting the genome in nearly independent blocks of linkage disequilibrium. Due to the complexity of this problem, these algorithms rely on heuristics, which makes them suboptimal.

**Results:**

Here, we develop an optimal solution for this problem using dynamic programming.

**Availability:**

This is now implemented as function snp_ldsplit as part of R package bigsnpr.

**Supplementary information:**

Supplementary data are available at *Bioinformatics* online.

## Introduction

A few algorithms have been developed for splitting the genome in nearly independent blocks of linkage disequilibrium (Berisa and Pickrell, 2016; Kim *et al.*, 2018). Dividing the genome in multiple smaller blocks has many applications. One application is to report signals from independent regions of the genome (Berisa and Pickrell, 2016; Ruderfer *et al.*, 2018; Wen *et al.*, 2017). Another application is for the development of statistical methods, e.g. for deriving polygenic scores (Ge *et al.*, 2019; Mak *et al.*, 2017; Zhou and Zhao, 2020), estimating genetic architecture and performing other statistical genetics analyses (Shi *et al.*, 2016; Wen *et al.*, 2016). Indeed, most statistical methods based on summary statistics also use a correlation matrix (between variants), and these methods often perform computationally expensive operations such as inversion and eigen decomposition of this correlation matrix. These operations are often quadratic, cubic or even exponential with the number of variants. However, if we can decompose the correlation matrix in nearly independent blocks, then we can apply these expensive operations to smaller matrices with less variants, making these operations much faster, and parallelizable. For instance, inverting a block-diagonal matrix requires only inverting each block separately.

## Implementation

**Fig. 1.**
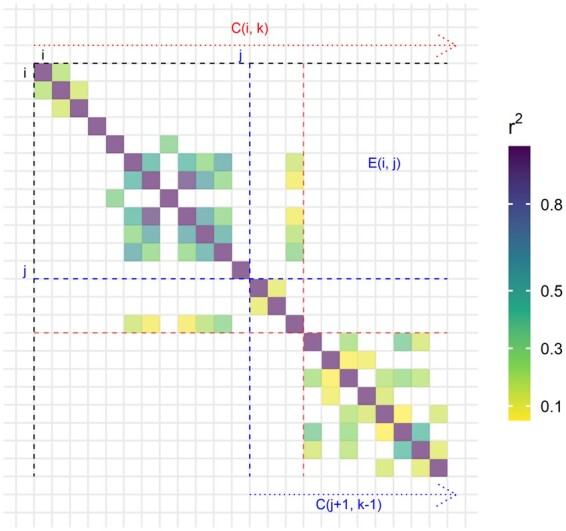
Illustration of subproblems solved by the algorithm using a small LD matrix. The cost of separating the region starting at variant *i* in *k* blocks exactly, *C*(*i*, *k*), is broken down in two: the error *E*(*i*, *j*), the sum of all squared correlations between variants from block (*i*, *j*) and variants from all the later blocks, and the cost of separating the rest starting at (j+1) using (k−1) blocks. The variant *j* at which the split occurs is chosen so that the cost (E(i,j)+C(j+1,k−1)) is minimized. The optimal split is highlighted in red here.

We aim at optimally splitting the genome into *K* blocks, where each block has a bounded number of variants (minimum and maximum size). This splitting is optimal in the sense that it minimizes the sum of squared correlations between variants from different blocks (hereinafter denoted as ‘cost’). This problem is quite complex, and a naive implementation would be exponential with the number of variants. To solve this problem efficiently, we use dynamic programming, which consists in breaking a problem into subproblems and then recursively finding the optimal solutions to the subproblems. Dynamic programming has been successfully used before to solve related problems such as haplotype block partitioning (Zhang *et al.*, 2002). Here, each subproblem consists in solving

(1) $$C(i,k)=\min_{j}\left\{E(i,j)+C(j+1,k-1)\right\},$$

where *C*(*i*, *k*) is the minimum cost for splitting the region from variant *i* to the last variant into *k* blocks exactly, and *E*(*i*, *j*) is the error/cost between block (*i*, *j*) and the latter blocks. This is illustrated in Figure 1. These subproblems can be solved efficiently by starting with *k *=* *1 and with *i* from the end of the region, and working our way up. Once all costs in the *C* matrix have been computed, and corresponding splits *j* have been recorded, the optimal split can be reconstructed from *C*(1, *K*), where *K* is the number of blocks desired. To efficiently compute $E(i,j)=\sum_{p=i}^{j}\sum_{q=j+1}^{m}R{\left(p,q\right)}^{2}$, where *m* is the number of variants and *R*(*p*, *q*) is the correlation between variants *p* and *q*, we first compute the matrix *L* defined as $L(i,j)=\sum_{q=j+1}^{m}R{\left(i,q\right)}^{2}$. Matrices *L* and *E* are sparse. *E* is the largest matrix and requires approximately m×(max_size-min_size)×4 bytes to be stored efficiently. For *m *=* *100 000, min_size = 500 and max_size = 10 000, this represents 3.5 GB. A description of the parameters of function snp_ldsplit implementing this method can be found in Supplementary section ‘Parameters of snp_ldsplit’.

## Results

As input, function snp_ldsplit uses a correlation matrix in sparse format from R package Matrix, which can be computed using the available snp_cor function from R package bigsnpr (Privé *et al.*, 2018). This function is fast and parallelized. Then, to run snp_ldsplit using a correlation matrix for 102 451 variants from chromosome 1, it takes <6 min on a laptop to find the optimal split in *K* blocks (for all *K *=* *1 to 133) with a bounded block size between 500 and 10 000 variants. Then, the user can choose the desired number of blocks, which is a compromise between having more (smaller) blocks with a higher overall cost (LD between blocks), and having less (larger) blocks with a smaller cost. For chromosome 1 and Europeans, ldetect report 133 linkage disequilibrium (LD) blocks (Berisa and Pickrell, 2016); however, we find that they can hardly be considered truly independent given the high cost (10 600) of the corresponding split (Supplementary Fig. S1). When splitting chromosome 1 for Europeans using the optimal algorithm we propose here, it can be split into 39 blocks at a cost of 1, in 65 blocks at a cost of 10, and in 133 blocks at a cost of 296 (Supplementary Fig. S1). Similar results are found for other chromosomes, and for Africans and Asians; however, splitting the LD from admixed Americans comes at a high cost (Supplementary Figs S2–S5). Both methods largely pick block boundaries at recombination hotspots (Supplementary Figs S7 and S8). We also provide an application to LD score regression in Supplementary section ‘Application to LD score regression’, where we show that standard errors for the SNP heritability using nearly independent blocks tend to be larger than when there is substantial LD between blocks, especially for phenotypes with large associations in the HLA (human leukocyte antigen) region (a long-range LD region).

## Software, code and data availability

The newest version of R package bigsnpr can be installed from GitHub (see https://github.com/privefl/bigsnpr). All code used for this article is available at https://github.com/privefl/paper-ldsplit/tree/master/code. The HapMap3 variants annotated with 242 blocks can be downloaded at https://www.dropbox.com/s/hdui60p9ohyhvv5/map_blocks.rds?dl=1. LD score regression results are available at https://github.com/privefl/paper-ldsplit/tree/main/ldsc_blocks, with a description of the 245 phenotypes used at https://github.com/privefl/UKBB-PGS/blob/main/phenotype-description.xlsx.

## Supplementary Material

btab519_Supplementary_Data
