# Supplementary material for: Optimal linkage disequilibrium splitting
Source: Bioinformatics. 2021 Jul 14;38(1):255–6. doi: 10.1093/bioinformatics/btab519 (PMC8696101; doi:10.1093/bioinformatics/btab519)
Supplement: btab519_Supplementary_Data [file btab519_supplementary_data.pdf]

Supplementary Materials

Parameters of snp_ldsplit

Function snp_ldsplit has five parameters:

- corr: A sparse correlation matrix, usually the output of snp_cor.
- thr_r2: Threshold under which squared correlations are ignored. This is useful to avoid counting noise, which should give clearer patterns of costs vs. number of blocks. It is therefore possible to have a splitting cost of 0 (a threshold of 5% is used in this paper). If this parameter is used, then corr can be computed using the same parameter in snp_cor (to increase the sparsity of the resulting matrix).
- min_size: Minimum number of variants in each block. This is used not to have a disproportionate number of small blocks.
- max_size: Maximum number of variants in each block. This is used not have blocks that are too large, e.g. to limit computational and memory requirements of applications that would use these blocks. For some long-range LD regions, it may be needed to allow for large blocks.
- max_K: Maximum number of blocks to consider. All optimal solutions for K from 1 to max_K will be returned. Some of these K might not have any corresponding solution due to the limitations in size of the blocks. For example, splitting 10,000 variants in blocks with at least 500 and at most 2000 variants implies that there are at least 5 and at most 20 blocks. Then, the choice of K depends on the application, but a simple solution is to choose the largest K for which the cost is lower than some threshold (see e.g. figure S9).

Procedure to compute optimal costs

We use the 1000 Genomes (1000G) data in the '.bed' PLINK format provided in Privé *et al.* (2020a), and composed of 2490 individuals and 1,664,852 variants. In turn, for individuals in each of the five super populations from the 1000G data, we compute the correlation between variants with MAF > 0.05 using function `snp_cor` from R package bigsnpr (Privé *et al.* 2018), assuming that correlations between variants more than 3 cM away are 0 (Privé *et al.* 2020b). Then, optimal splits are computed using function `snp_ldsplit` introduced in this paper, setting parameters `thr_r2 = 0.05`, `min_size = 500`, and `max_size = 10000`. These costs are compared with the costs provided by ldetect (Berisa and Pickrell 2016) for the European and African populations only since they are not provided for the other three super-populations. Results are presented in figures S1-S5. We see that splitting costs are very high for the admixed Americans group, showing that it is impossible to split LD in independent blocks in an admixed population. This is also the case, to a lesser extent, for the African (AFR) group which includes African Americans (ASW) and African Caribbeans (ACB) who may be admixed with e.g. European ancestry. When excluding 5% of AFR individuals based on principal component analysis, splitting costs are reduced (Figure S6).

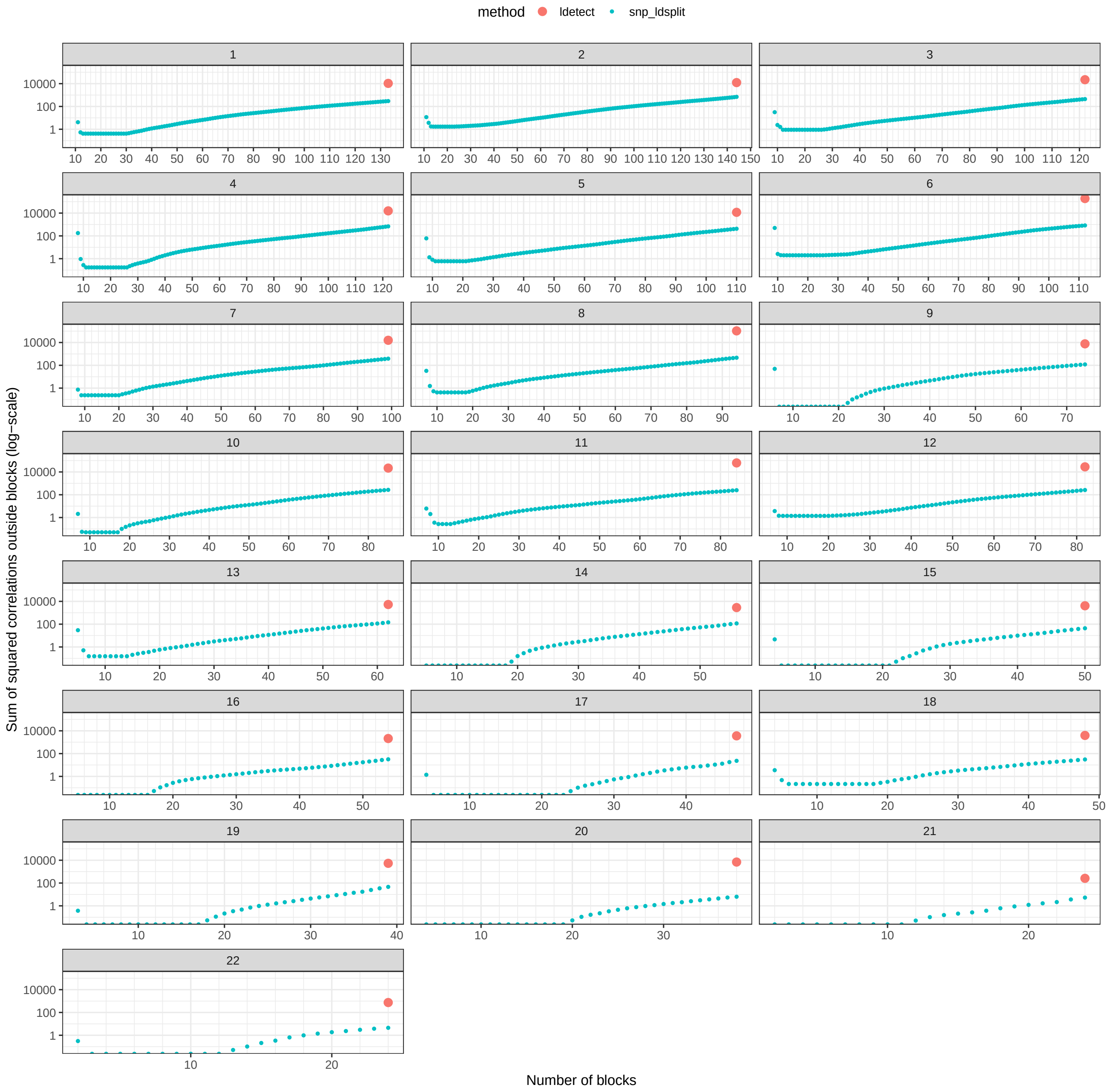

Figure S1: Optimal costs of splitting chromosomes (each panel) in a specified number of blocks (including 500 variants at minimum and 10,000 at maximum) for the European (**EUR**) super-population in the 1000 Genomes data. The cost is defined as the sum of squared correlations r^2 between variants from different blocks, restricting to all $r^2 > 0.05$. In red is the cost from the split from ldetect, accessed from `https://bitbucket.org/nygcresearch/ldetect-data/src/master/EUR/`.

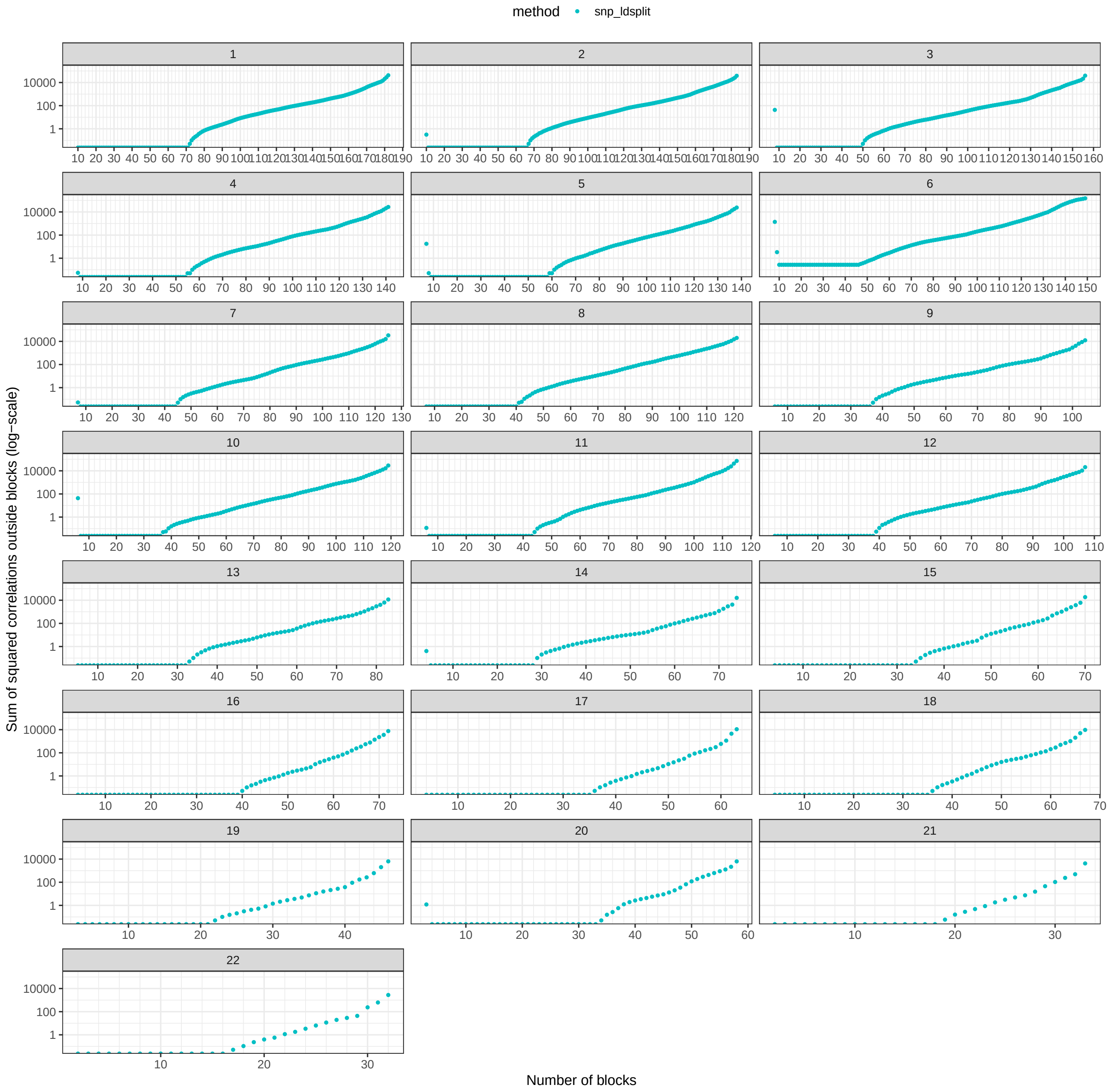

Figure S2: Optimal costs of splitting chromosomes (each panel) in a specified number of blocks (including 500 variants at minimum and 10,000 at maximum) for the East Asian (**EAS**) super-population in the 1000 Genomes data. The cost is defined as the sum of squared correlations r^2 between variants from different blocks, restricting to all $r^2 > 0.05$.

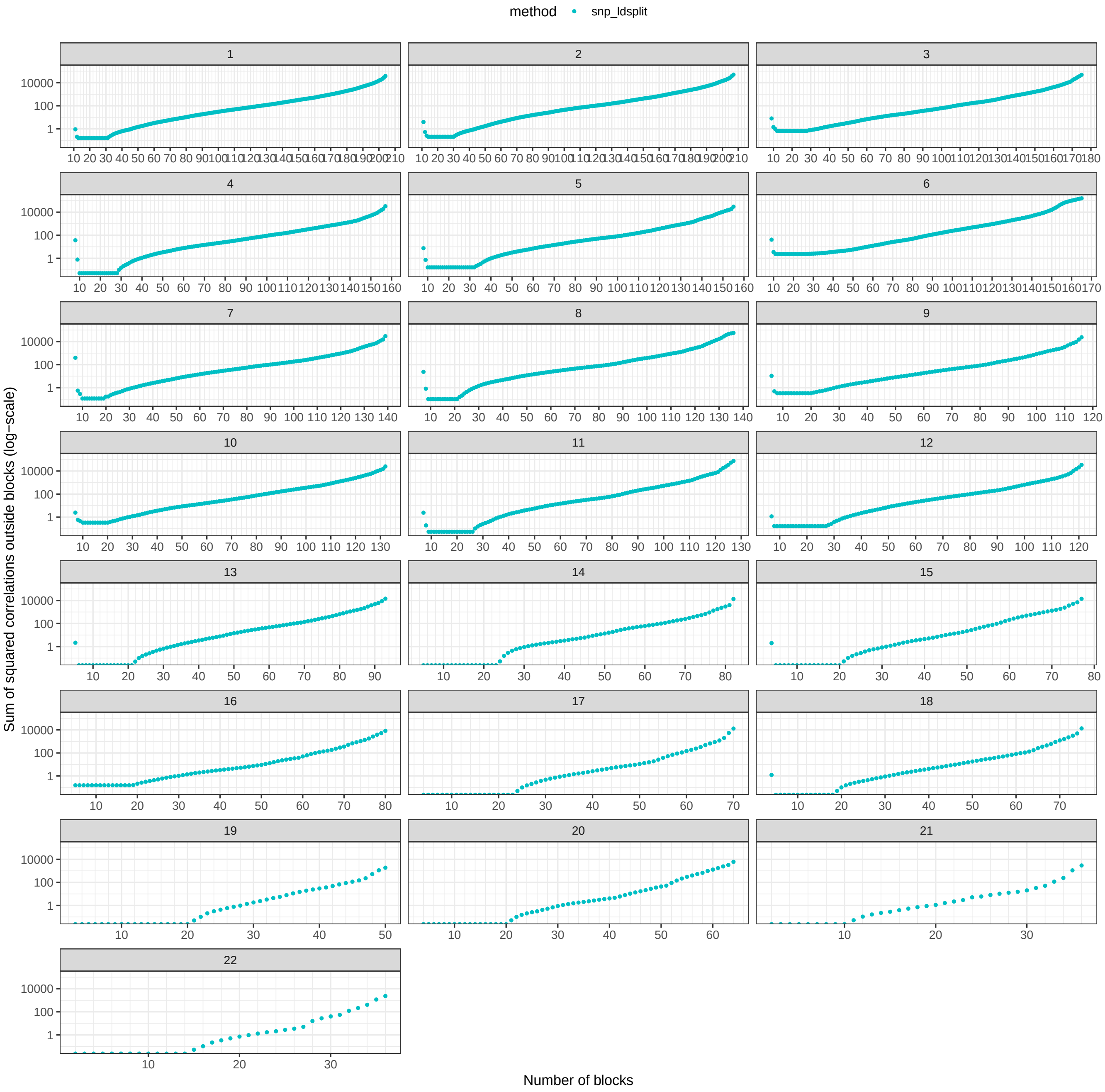

Figure S3: Optimal costs of splitting chromosomes (each panel) in a specified number of blocks (including 500 variants at minimum and 10,000 at maximum) for the South Asian (**SAS**) super-population in the 1000 Genomes data. The cost is defined as the sum of squared correlations r^2 between variants from different blocks, restricting to all $r^2 > 0.05$.

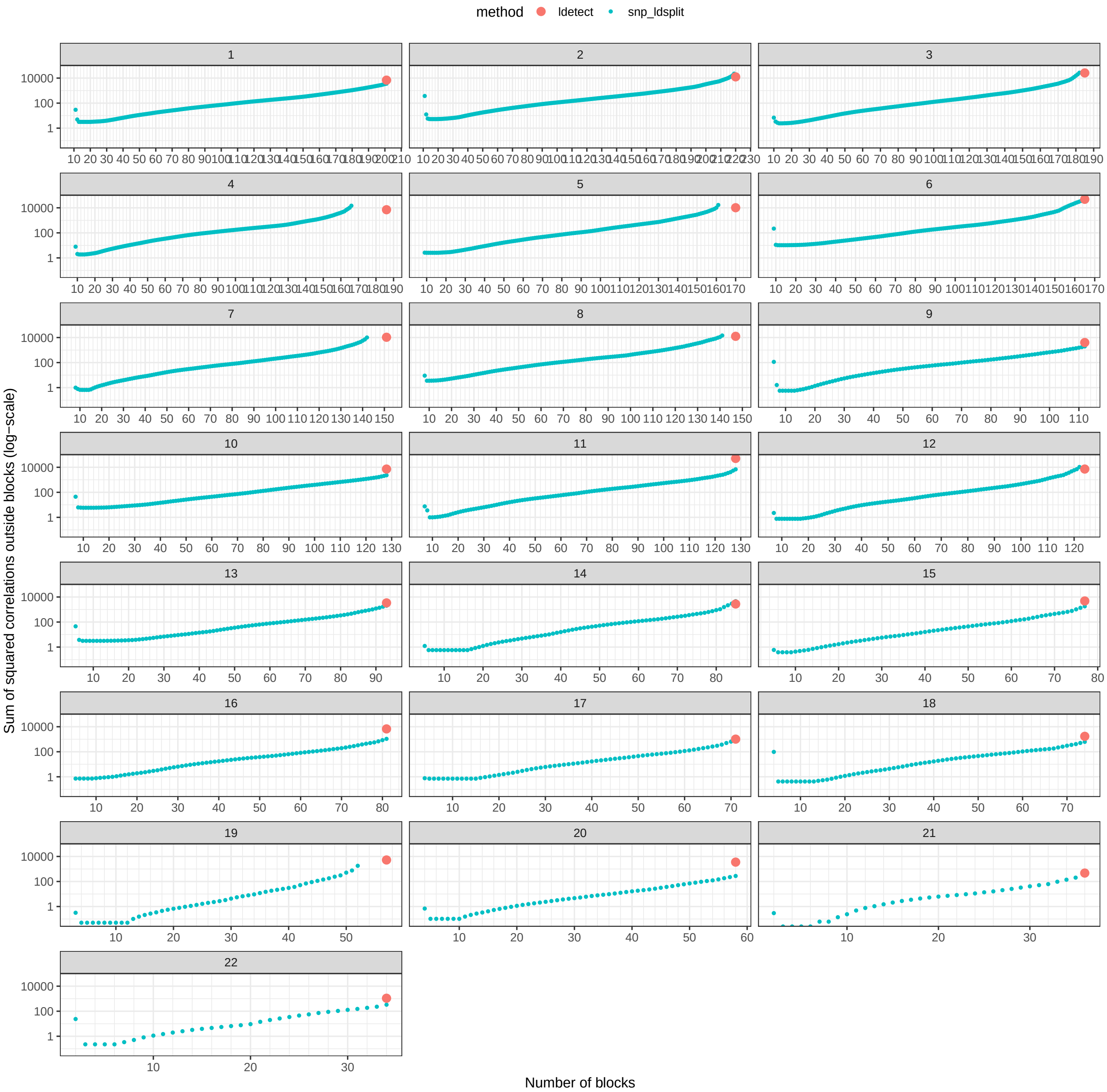

Figure S4: Optimal costs of splitting chromosomes (each panel) in a specified number of blocks (including 500 variants at minimum and 10,000 at maximum) for the African (**AFR**) super-population in the 1000 Genomes data. The cost is defined as the sum of squared correlations r^2 between variants from different blocks, restricting to all $r^2 > 0.05$. In red is the cost from the split from ldetect, accessed from `https://bitbucket.org/nygcresearch/ldetect-data/src/master/AFR/`. Here, for the same number of blocks, the cost using ldetect can be smaller than the one with our optimal splitting since we have an additional restriction that blocks should contain at least 500 variants.

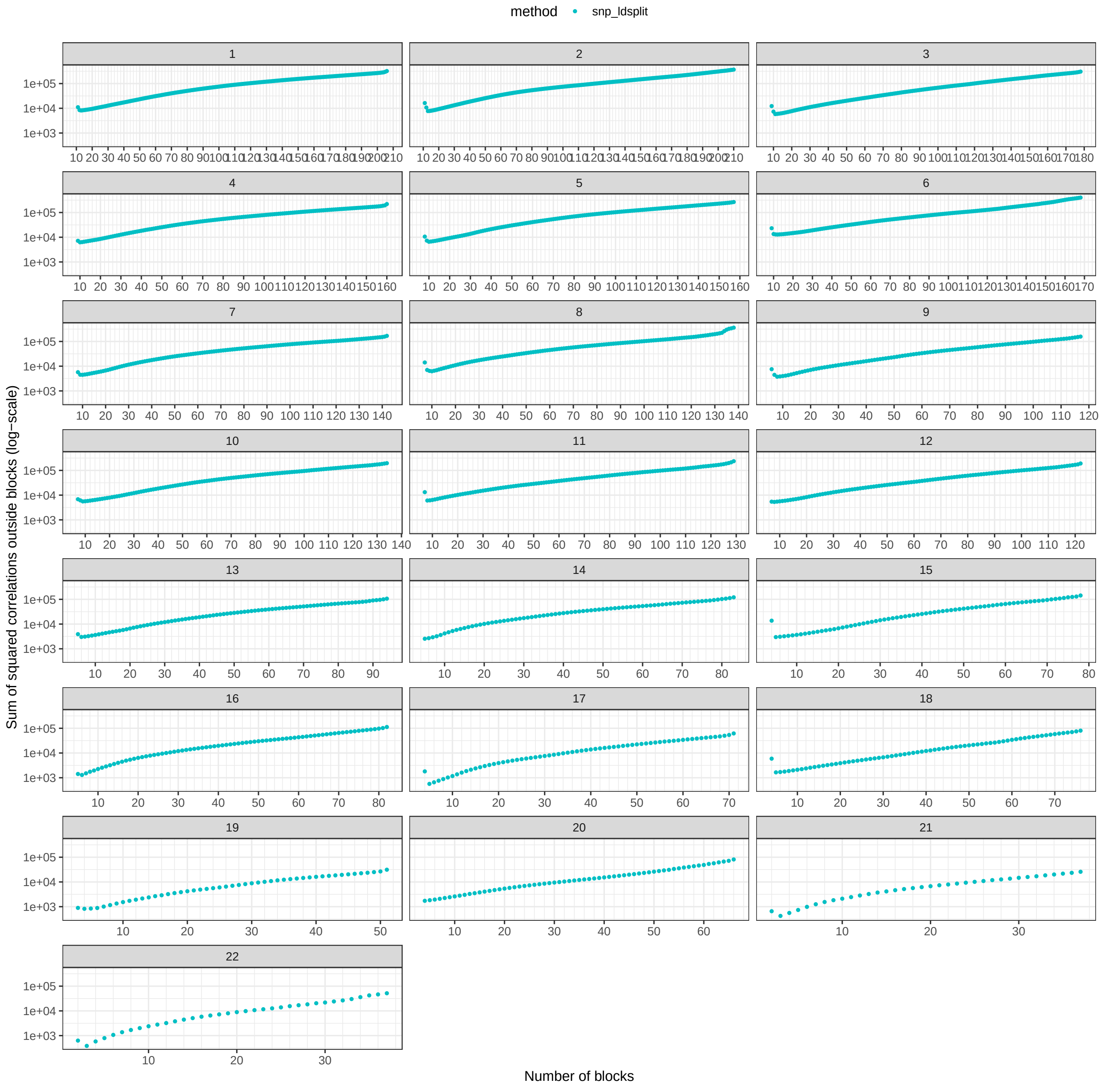

Figure S5: Optimal costs of splitting chromosomes (each panel) in a specified number of blocks (including 500 variants at minimum and 10,000 at maximum) for the Admixed American (**AMR**) super-population in the 1000 Genomes data. The cost is defined as the sum of squared correlations r^2 between variants from different blocks, restricting to all $r^2 > 0.05$.

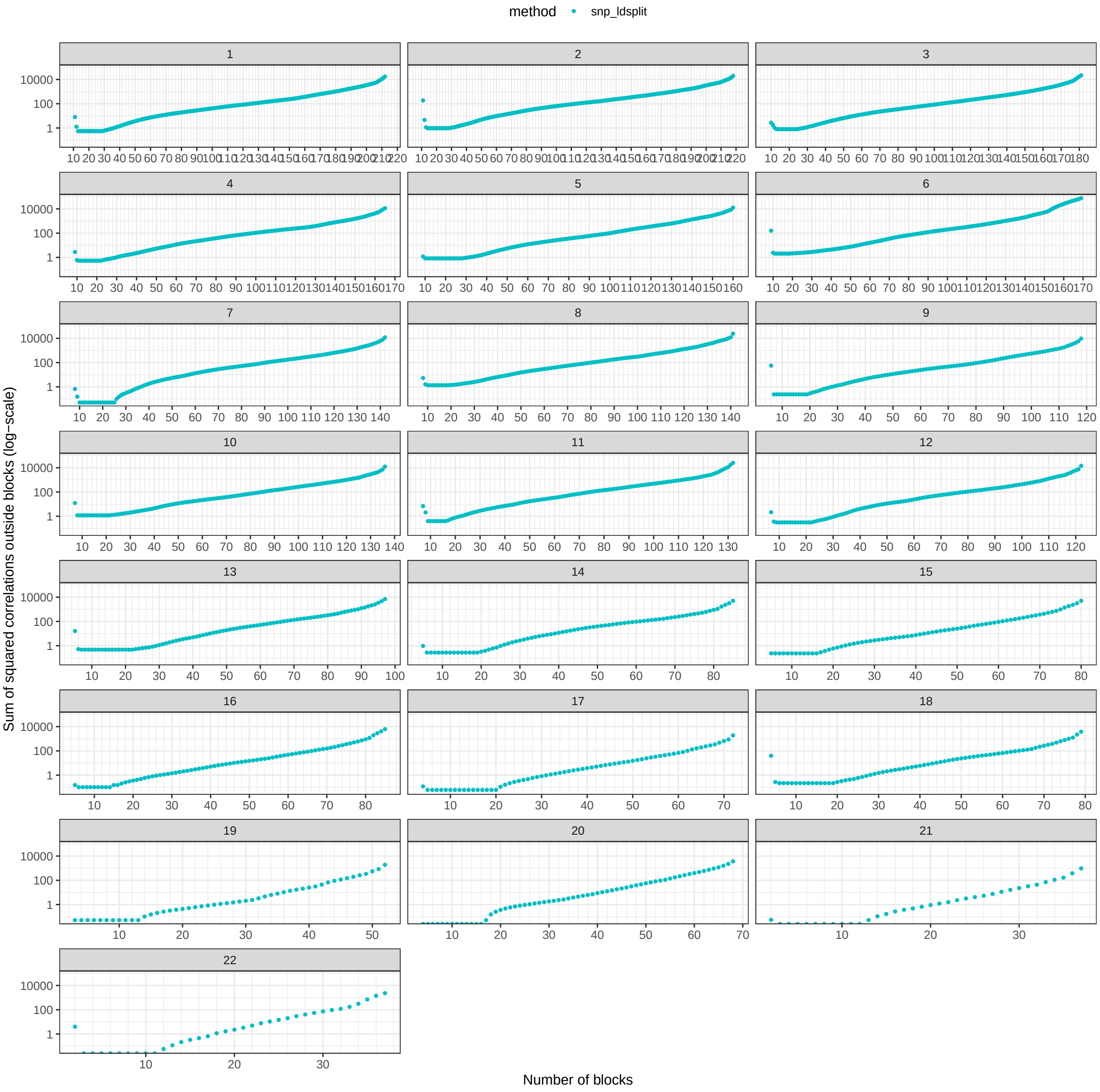

Figure S6: Same as figure S4, but after excluding 5% of AFR individuals based on PCA.

Comparison with recombination rates

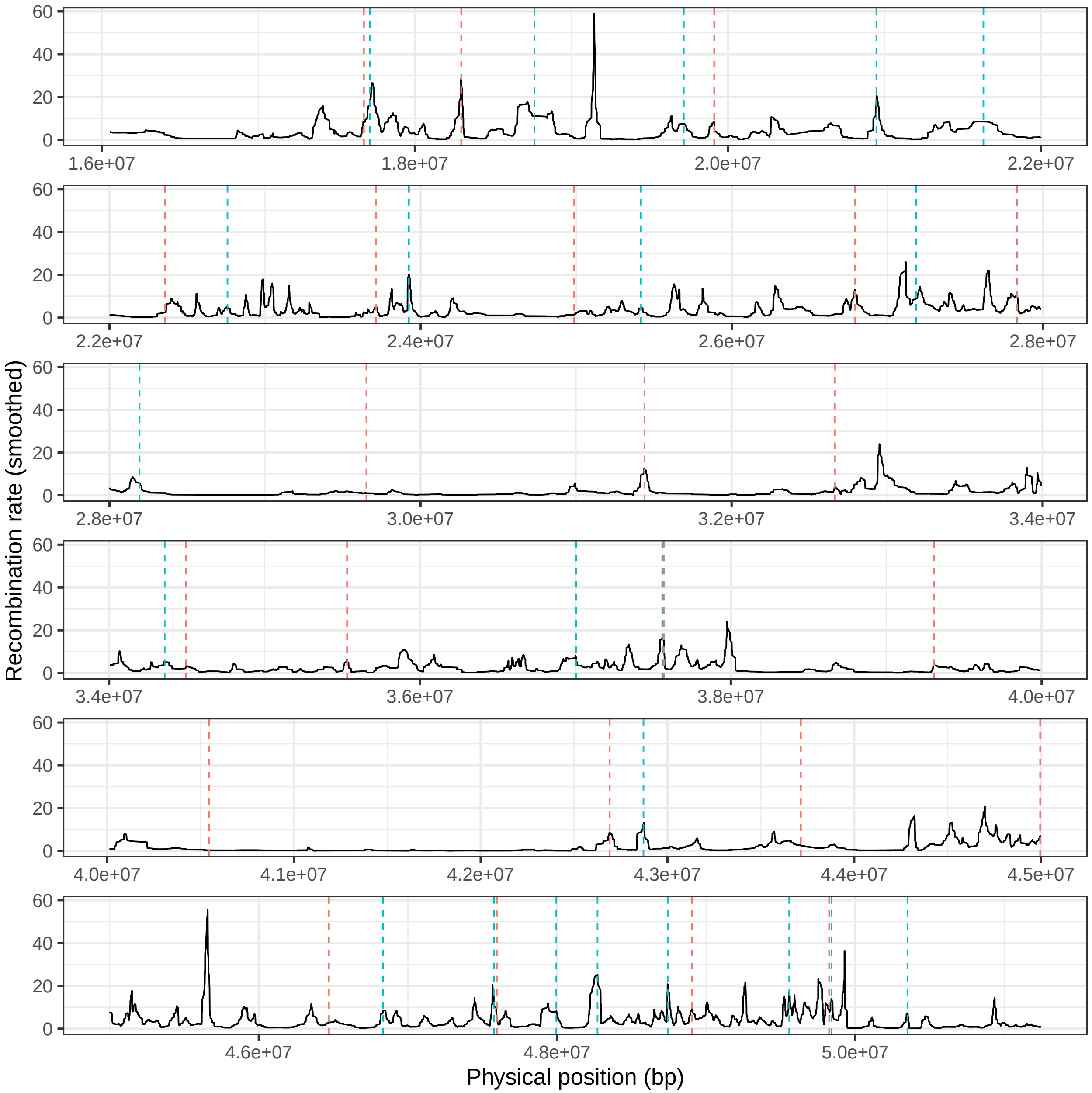

Figure S7: Recombination rates for chromosome 22 in the CEU population, as provided in Spence and Song (2019). Blue dotted lines correspond to boundaries of 22 blocks obtained using `snp_ldsplit`, while red dotted lines correspond to the ones from `ldetect`.

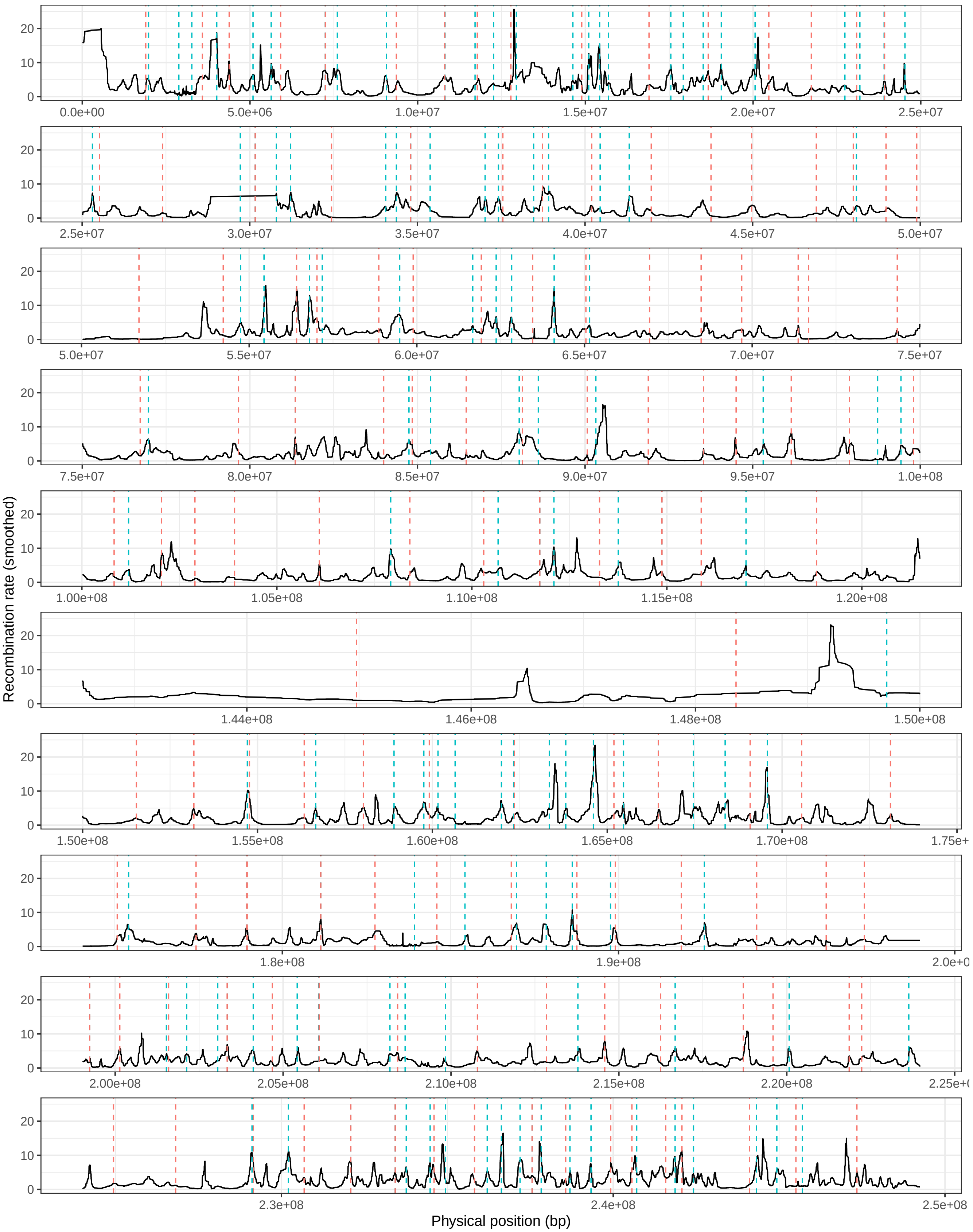

Figure S8: Recombination rates for chromosome 1 in the CEU population, as provided in Spence and Song (2019). Blue dotted lines correspond to boundaries of 133 blocks obtained using `snp_ldsplit`, while red dotted lines correspond to the ones from `ldetect`.

Application to LD score regression

LD score regression is a method to estimate confounding and SNP heritability from summary statistics (Bulik-Sullivan *et al.* 2015). By default, it uses the delete-a-group jackknife to compute standard errors associated with these two estimates by splitting the genome evenly into 200 blocks of contiguous variants. These blocks are not always independent, which is breaking one of the assumption of the jackknife. Here we aim at investigating the impact of using different blocks to compute standard errors in LD score regression. We use the function `snp_ldsplit` introduced in this paper with the LD reference of 1,054,330 HapMap3 variants provided in Privé *et al.* (2020b), and with parameters `thr_r2 = 0.05`, `min_size = 3000`, and `max_size = 10000`. Here, we set `min_size = 3000` not to have group sizes varying too much. For each chromosome, we choose the maximum K for which the optimal cost is zero, or very small in the case of chromosome 6 (Figure S9). This results in a total of 242 nearly-independent blocks. We also define another set of 243 blocks by randomly assigning approximately half of the variants of each group to the next group. This manipulation results in having new group boundaries at the middle of the 242 previous ones, introducing LD between neighboring blocks.

We use the implementation of LD score regression from R package bigsnpr (function `snp_ldsc`, Privé *et al.* (2018)), and modify it to allow for using the jackknife with groups of different sizes (Busing *et al.* 1999). For 245 phenotypes defined elsewhere (Privé *et al.* 2021), we run LD score regression either using the new set of nearly-independent blocks, or with the other set with LD between neighboring blocks. Standard errors using nearly-independent blocks tend to be larger than when there is substantial LD between blocks, especially for phenotypes with large associations in the HLA region (a long-range LD region), with e.g. standard errors of the SNP heritability estimate of 0.00378 vs. 0.00252 for lupus (phecode 695.4), 0.0302 vs. 0.0205 for celiac disease (557.1), 0.00420 vs. 0.00304 for thyrotoxicosis (242), 0.00313 vs. 0.00230 for sicca syndrome (709.2), and 0.00385 vs. 0.00294 for hematuria (593).

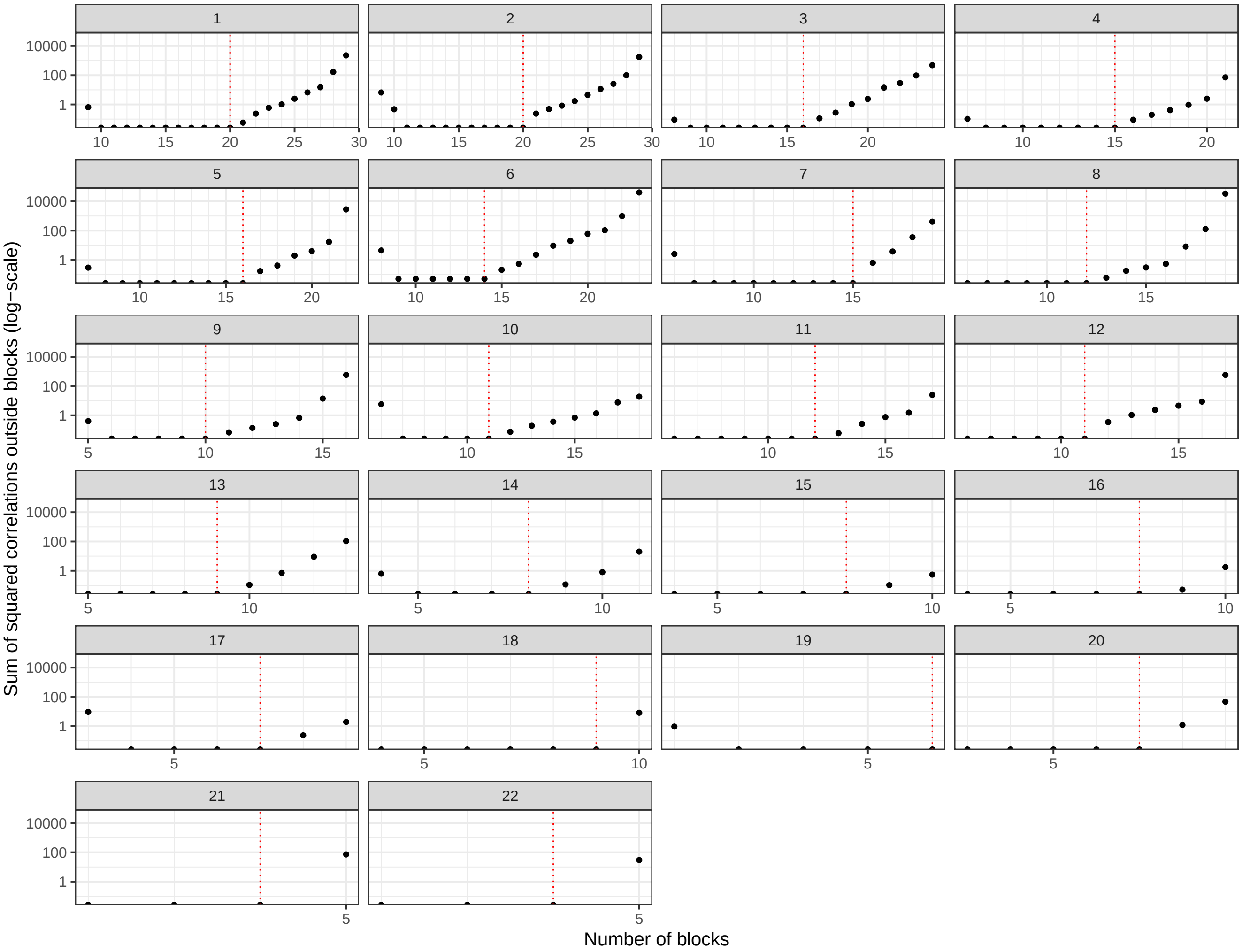

Figure S9: Optimal costs of splitting chromosomes (each panel) in a specified number of blocks (including 3000 variants at minimum and 10,000 at maximum) for the European LD reference provided in Privé *et al.* (2020b). Red dotted lines highlight the number of blocks chosen for each chromosome. The cost is defined as the sum of squared correlations r^2 between variants from different blocks, restricting to all $r^2 > 0.05$.

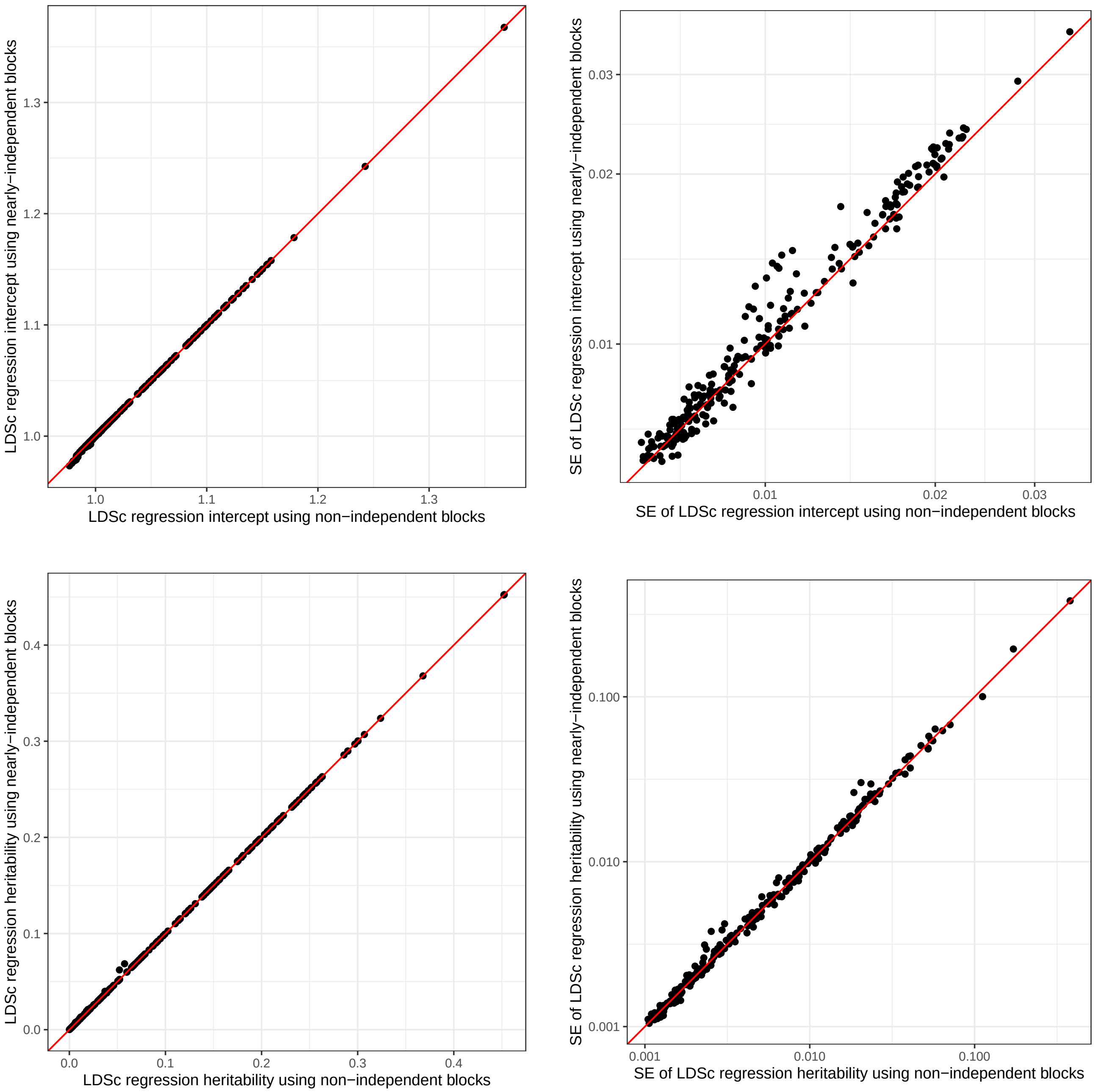

Figure S10: LD score regression results for 245 phenotypes, when using either nearly-independent blocks for jackknifing, or blocks with some LD between neighboring blocks. “SE” means standard error, and are represented using a log scale.

References

Berisa, T. and Pickrell, J. K. (2016). Approximately independent linkage disequilibrium blocks in human populations. *Bioinformatics*, **32**(2), 283.

Bulik-Sullivan, B. K., Loh, P.-R., Finucane, H. K., Ripke, S., Yang, J., Patterson, N., Daly, M. J., Price, A. L., and Neale, B. M. (2015). LD Score regression distinguishes confounding from polygenicity in genome-wide association studies. *Nature Genetics*, **47**(3), 291–295.

Busing, F. M., Meijer, E., and Van Der Leeden, R. (1999). Delete-m jackknife for unequal m. *Statistics and Computing*, **9**(1), 3–8.

Privé, F., Aschard, H., Ziyatdinov, A., and Blum, M. G. B. (2018). Efficient analysis of large-scale genome-wide data with two R packages: bigstatsr and bigsnpr. *Bioinformatics*, **34**(16), 2781–2787.

Privé, F., Luu, K., Blum, M. G., McGrath, J. J., and Vilhjálmsson, B. J. (2020a). Efficient toolkit implementing best practices for principal component analysis of population genetic data. *Bioinformatics*, **36**(16), 4449–4457.

Privé, F., Arbel, J., and Vilhjálmsson, B. J. (2020b). LDpred2: better, faster, stronger. *Bioinformatics*, **36**(22-23), 5424–5431.

Privé, F., Aschard, H., Carmi, S., Folkersen, L., Hoggart, C., O'Reilly, P. F., and Vilhjálmsson, B. J. (2021). High-resolution portability of 245 polygenic scores when derived and applied in the same cohort. *medRxiv*.

Spence, J. P. and Song, Y. S. (2019). Inference and analysis of population-specific fine-scale recombination maps across 26 diverse human populations. *Science advances*, **5**(10), eaaw9206.
